# A Novel BRD Family PROTAC Inhibitor dBET1 Exerts Great Anti-Cancer Effects by Targeting c-MYC in Acute Myeloid Leukemia Cells

**DOI:** 10.3389/pore.2022.1610447

**Published:** 2022-06-27

**Authors:** Kunlong Zhang, Li Gao, Jianwei Wang, Xinran Chu, Zimu Zhang, Yongping Zhang, Fang Fang, Yanfang Tao, Xiaolu Li, Yuanyuan Tian, Zhiheng Li, Xu Sang, Li Ma, Lihui Lu, Yanling Chen, Juanjuan Yu, Ran Zhuo, Shuiyan Wu, Jian Pan, Shaoyan Hu

**Affiliations:** ^1^ Children’s Hospital of Soochow University, Suzhou, China; ^2^ Department of Pediatrics, The Second Hospital of Anhui Medical University, Hefei, China; ^3^ Department of Hematology, Children’s Hospital of Soochow University, Suzhou, China; ^4^ Institute of Pediatric Research, Children’s Hospital of Soochow University, Suzhou, China; ^5^ Intensive Care Unit, Children’s Hospital of Soochow University, Suzhou, China

**Keywords:** acute myeloid leukemia, C-MYC, BRD4, dBET1, PROTAC

## Abstract

Acute myeloid leukemia (AML) represents an aggressive hematopoietic malignancy with a prognosis inferior to that of other leukemias. Recent targeted therapies offer new opportunities to achieve better treatment outcomes. However, due to the complex heterogeneity of AML, its prognosis remains dismal. In this study, we first identified the correlation between high expression of BRD4 and overall survival of patients with AML. Targeted degradation of BRD2, BRD3, and BRD4 proteins by dBET1, a proteolysis-targeting chimera (PROTAC) against the bromodomain and extra-terminal domain (BET) family members, showed cytotoxic effects on Kasumi (AML1-ETO), NB4 (PML-RARa), THP-1 (MLL-AF9), and MV4-11 (MLL-AF4) AML cell lines representing different molecular subtypes of AML. Furthermore, we determined that dBET1 treatment arrested cell cycling and enhanced apoptosis and c-MYC was identified as the downstream target. Collectively, our results indicated that dBET1 had broad anti-cancer effects on AML cell lines with different molecular lesions and provided more benefits to patients with AML.

## Introduction

Acute myeloid leukemia (AML) is an aggressive hematopoietic cancer characterized by blocked differentiation of myeloid lineage [1-3]. Due to risk stratification-directed therapy, treatment outcomes with a 5-year survival rates of 40%–45% in younger patients (<60 years) and 10%–15% in older patients (>60 years) have improved in the past decades[1, 2]. However, except for acute promyelocytic leukemia (APL) and core-binding factor (CBF) AML, prognosis remains dismal with conventional chemotherapy (3 + 7 regimen: 3 days of daunorubicin + 7 days of cytarabine), which might have reached the limit for treating AML [4-7]. Therefore, novel therapies are urgently needed to achieve better treatment outcomes for patients with AML, especially those who are older.

AML is highly genetically heterogenous. Cytogenetic analysis revealed major mutations, including chromatin translocation, deletions, insertions, and inversions, among others, in approximately 55% of AML cases. Based on cytogenetic analyses, patients with AML were classified into the favorable, intermediate, and unfavorable groups. More recently, progress in genetics of AML has discovered several recurrent mutations in the remaining 45% of patients with AML, with being prognostic factors (i.e., *NPM1*, *FLT3*, *IDH1*/*2*, *KIT*, *BCL-2*, *TP53*, and *MDM2*) [8-13]. Moreover, specific inhibitors have been developed to target these mutations. For instance, several FLT3 inhibitors (e.g., Midostaurin, Gilteritinib, and Quizartinib) have been approved or been in clinical trials as single-agent therapy or being components of combination therapy [14, 15]. These targeted therapies have led to improved overall survival (OS) of patients with AML with certain genetic mutations. However, drug-resistance and off-target effects emerged as one of the challenges in using these small-molecular inhibitors. Furthermore, the genetic complexity of AML has imposed challenges to translate these molecular lesions into effective targeted therapies to provide more benefits to patients with AML. More targets with therapeutic potential must be discovered.

The BET family members (e.g., BRD2, BRD3, BRD4, and BRDT) are epigenetic readers that recognize acetylated proteins, including histone and nonhistone proteins (e.g., transcription factors) [16-18]. The versatile functions of the BET family have been implicated in RNA translation, epigenetic modifications and transcriptional regulation among many other roles in cell proliferation, apoptosis, and immune response [19, 20]. In human cancers, BET family members have been proven to be essential for the survival of several types of tumor cells [21-24]. Especially in AML, RNA interference screening suggested BRD4 as a vulnerability in AML. Moreover, inhibition of BRD4 by JQ1, a small-molecular inhibitor of BRD4, showed robust antileukemic activity potentially by suppression of MYC both *in vitro* and *in vivo* [23]. Furthermore, subsequent preclinical studies proved the therapeutic potential of BET inhibitors in different cancers [19, 23, 25].

Recently, a new technology, proteolysis-targeting chimera (PROTAC), has been developed, consisting of a ligand for a protein of interest, a linker, and an adaptor to recruit an E3 ubiquitin ligase. A PROTAC would bring its target protein in close proximity to a E3 ligase, and initiate polyubiquitination and subsequent proteasome-mediated degradation [26]. There are several challenges toward the application of traditional small-molecular inhibitors, including drug selectivity, therapy resistance, and unavailability of undruggable proteins. However, PROTACs would overcome these challenges [27]. dBET1, a PROTAC, generated based on JQ1, has been developed to target BRD2, BRD3 and BRD4, and its efficacy has been evaluated in the AML cell line, MV4-11 [28]. AML is a genetically complex disease, composed of different molecular subtypes [29], however, the cytotoxic effects of dBET1 on other subtypes remain poorly understood.

In this study, we explored the cytotoxic effects of dBET1 on several AML cell lines (i.e., NB4, Kasumi, THP-1, and MV4-11), representing different molecular subtypes (i.e., PML-RARa, AML1-ETO, MLL-AF4, and MLL-AF9, respectively) of AML. Moreover, we identified the inhibition of cell cycling and enhancing apoptosis as being the downstream effector of the degradation of BRD2, BRD3, and BRD4 by dBET1. Our results shed new light on the molecular mechanism of dBET1’s cytotoxicity and expanded the therapeutic potential of dBET1 to more molecular subtypes of AML.

## Materials and Methods

### Cell Culture

NB4, MV4-11, Kasumi, and THP-1 cells were purchased from National Collection of Authenticated Cell Cultures and were grown in culture in RPMI 1640 medium (GIBCO, Life Technologies) supplemented with 10% heat-inactivated FBS, 2 mM L-glutamine, 100 units/ml penicillin G sodium, and 100 μg/ml streptomycin sulfate at 37°C in 5% CO_2_.

### Public Data

Public expressions data of BRD4 in 1389 cell lines was downloaded from DepMap (https://depmap.org/portal/) and shown as a box plot by different tissue/organ. Survival curves of adult patients with AML from TCGA with high/low expression of BRD4/c-MYC were generated from GEPIA2 (http://gepia2.cancer-pku.cn/#index).

### Cell Viability Assay

AML cell lines were plated at a density of 2 × 10^4^ cells per well in 96-well plate and treated with variable concentrations of dBET1(cat. HY-101838; MedChemExpress). After 48 h of treatment, cell viability was tested with Cell Counting Kit-8 (CCK8) kit (Dojindo Molecular Technologies). Absorbance at 450 nm was applied to estimate the cell viability, which was normalized to DMSO control. Cell viability assay was performed in triplicate, and each assay was repeated at least three times. The half-maximal inhibitory concentration (IC_50_) of dBET1 was calculated using Graph Prism version 8 (GraphPad Software, Inc., San Diego, CA, United States).

### Colony Formation Assay

NB4, MV4-11, Kasumi, and THP-1 cells were seeded with the same density (4 × 10^3^ cells per well) in six-well plate of soft agar supplemented with variable concentrations of dBET1. After 10 days, cells were first fixed with 100% methanol for 15 min, followed by Giemsa staining for 1 h. Images were acquired using an optical microscope, and the number of colonies were manually counted.

### Lentiviral Infection

Short hairpin RNA (shRNA) targeting CRBN (the sequences: CCG​GGC​CCA​CGA​ATA​GTT​GTC​ATT​TCT​CGA​GAA​ATG​ACA​ACT​ATT​CG) was cloned into pLKO.1 plasmid [30], and cDNA sequence of CRBN tagged with V5 was cloned into pLX304 plasmid [31] (a gift from Dr. X. Liang at the Cancer Science Institute of Singapore). pMD2.G and psPAX2 were used for packaging lentivirus in 293T cells. Twenty hours after incubation with virus supernatant, cells were subjected to puromycin (Cat.#ST551, Beyotime, China) selection (10 μg/ml).

### RNA-Seq and Data Analysis

Total RNA was extracted using the RNeasy Mini kit (cat. 74104; Qiagen, Germany). Library preparation, transcriptome sequencing (Illumina NovaSeq 6000) and clean data filtering were carried out by Novogene Bioinformatics Technology Co., Ltd. (Beijing, China). The 150 bp paired-end reads were aligned to hg38 (Ensembl) genome using HISAT2 software (version 2.2.0). Transcriptome assembly and abundance analysis were performed with StringTie software (version 2.1.2). Differentially expressed genes were identified by R/Bioconductor package DESeq2 with adjusted *p* value <0.05 and absolute log2 (fold change) > 1. Gene Ontology (GO) and gene set enrichment analysis (GSEA) with Hallmarks gene sets (version 7.1) were conducted using R/Bioconductor package cluster Profiler [32]. Heatmaps of genes were generated by R/Bioconductor package pheatmap. RNA-seq data supporting the results of current study have been deposited in GEO database (https://www.ncbi.nlm.nih.gov/geo/query/acc.cgi?acc=GSE196693). The data can be accessible with a token upon request.

### Cell Cycle Analysis

NB4, MV4-11, Kasumi, and THP-1 cells were treated with dBET1 for 24 h and were then fixed with 70% ethanol and incubated at 4°C for overnight, followed by washing with cold 1× phosphate-buffered saline (PBS) buffer. Fixed cells were then incubated with 1.5 μM propidium iodide (PI) (Sigma-Aldrich, St. Louis, MO, United States) solution supplemented with RNase A (25 μg/ml) at room temperature for 1 h. Cell cycle distribution was determined using flow cytometry and analyzed using MultiCycle AV DNA analysis software (Verity Software House, Topsham, ME, United States).

### Cell Apoptosis Assay

Cell apoptosis was analyzed as previously described [33]. Briefly, NB4, MV4-11, Kasumi, and THP-1 cells were treated with dBET1 at indicated concentrations. After 24 h incubation, cells were harvested and washed with cold 1 × PBS buffer. Cells were then suspended in the 1× binding buffer and stained with FITC-Annexin V antibody and PI solution according to the manual of the FITC-Annexin V apoptosis kit (cat. 556420; BD Biosciences, Franklin Lakes, NJ, United States). Cell apoptosis was analyzed by Beckman Gallios™ Flow Cytometer (Beckman, Krefeld, Germany).

### Quantitative Real-Time Polymerase Chain Reaction

Total RNA was purified using the RNeasy Mini Kit (cat. 74104; Qiagen, Germany). First-strand cDNA was generated using a mix of 2 μg of total RNA, 500 ng of random primers (Promega, United States), 200U of M-MLV reverse transcriptase (Promega, United States), and 20U of RNase inhibitor (Thermo Fisher Scientific, MA, United States) in a total volume of 20 μl. qRT-PCR was performed using LightCycler® 480 SYBR Green I Master mix (cat. 04707516001; Roche, Penzberg, Germany) on a Light cycler 480 Real-Time System (Roche, Penzberg, Germany) according to the standard protocol. Quantitative mRNA expression of MYC (forward: ATG​GCC​CAT​TAC​AAA​GCC​G, reverse: TTT​CTG​GAG​TAG​CAG​CTC​CTA​A) was calculated using the Ct method using glyceraldehyde 3-phosphate dehydrogenase (GAPDH) expression (forward: TGC​ACC​ACC​AAC​TGC​TTA​G, reverse: GAT​GCA​GGG​ATG​ATG​TTC) as an internal reference.

### Western Blotting

**FIGURE 1:**
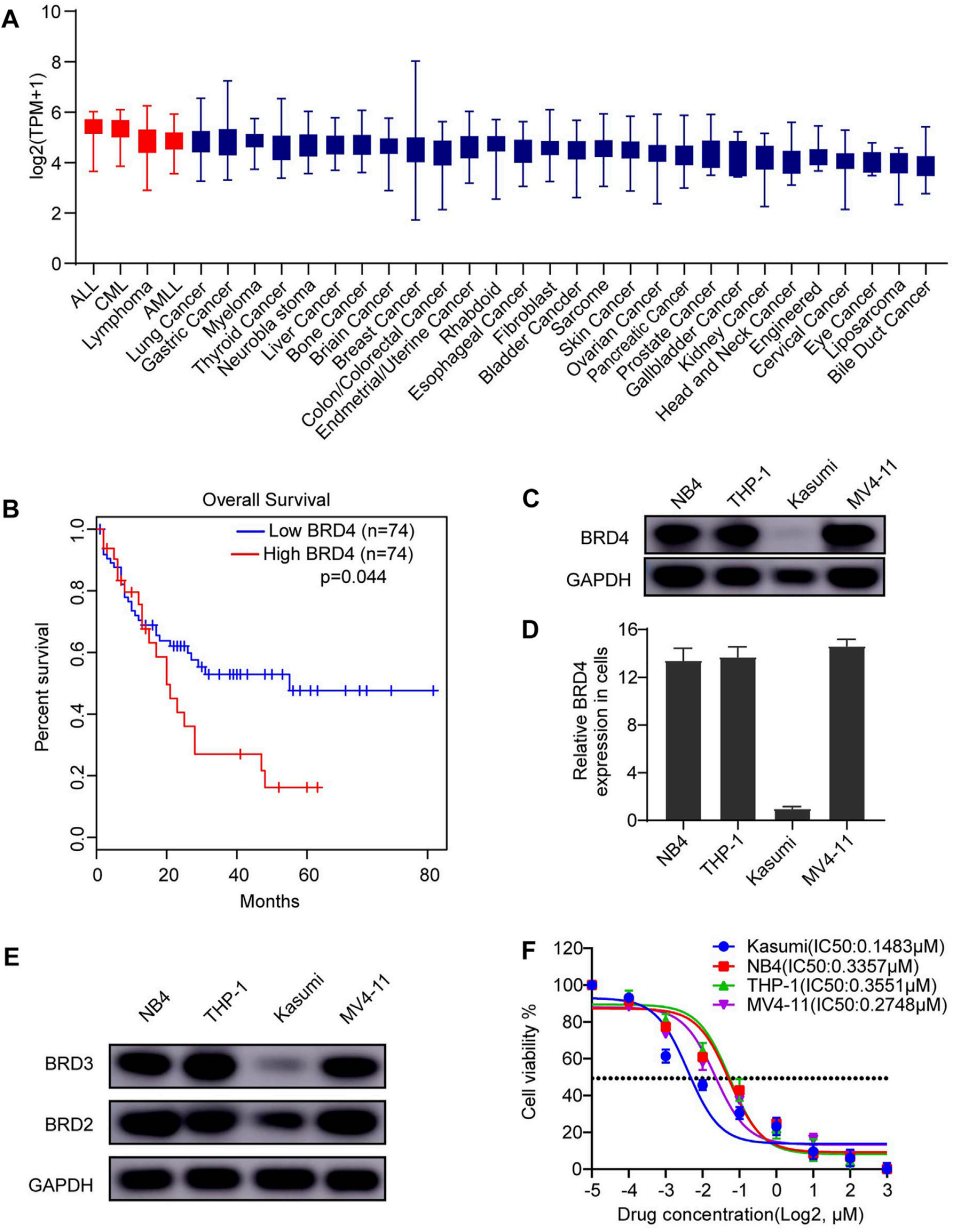
BRD4 is a promising therapeutic target for AML. **(A)** The expression of BRD4 was characterized in different cancer cell lines. High expression of BRD4 was observed in AML cell lines. **(B)** In a cohort of adult patient with AML (*n* = 148), patients with high expression of BRD4 was significantly associated with poorer overall survival (OS) (*p* = 0.044). **(C)** Expression of BRD4 was evaluated in four AML cell lines: NB4 (PML-RARa), Kasumi (AML1-ETO), THP-1 (MLL-AF9), and MV4-11 (MLL-AF4), cells, BRD4 was positively expressed in all cell lines observed using Western blot. **(D)** GAPDH served as an internal control. Densities of protein bands of BRD4 in each cell line were quantified by ImageJ and normalized to the densities of GAPDH bands. **(E)** Expressions of BRD2 and BRD3 were evaluated in the same four AML cell lines: NB4 (PML-RARa), Kasumi (AML1-ETO), THP-1 (MLL-AF9), and MV4-11 (MLL-AF4), cells, BRD2 and BRD3 were also positively expressed in all cell lines observed using Western blot. **(F)** The cytotoxic effects of dBET1 were determined using CCK-8 assay. The IC_50_ was estimated for each cell line.

Cells were lysed using RIPA buffer containing protease and phosphatase inhibitor cocktail (Roche, Penzberg, Germany) for 30 min on ice. The supernatant was collected by centrifugation as total protein and the concentration of total protein was quantified using the Pierce BCA Kit (Thermo Fisher Scientific). Blotting was performed as previously described [33]. Primary antibodies against the following proteins were used: BRD2(cat:5848s, 1:1,000, Cell Signaling Technology), BRD3(cat: 11859-1-AP, 1:1,000, Proteintech), BRD4 (cat: 13440s, 1:1,000, Cell Signaling Technology), CRBN (cat: HPA045910, Sigma-Aldrich), c-MYC (cat: 9402, 1:1,000, Cell Signaling Technology), PARP (Cat: 9542, 1:1,000, Cell Signaling Technology). GAPDH (1:2,000; MA3374, Millipore) was used as an internal control. The horseradish peroxidase (HRP)-conjugated secondary antibodies: Peroxidase AffiniPure Goat Anti-Mouse IgG(H+L) (cat: 115-035-003) and Goat Anti-Rabbit IgG(H+L) (cat: 111-035-003) were purchased from Jackson ImmunoResearch Laboratories, INC. The bands were visualized using an enhanced chemiluminescent (ECL) detection kit (Pierce, Rockford, IL, United States) using LAS 4010 (GE Healthcare Life Sciences, Little Chalfont, United Kingdom). Various concentrations (0, 0.5, 1.0 μM) of (R)-MG132 (cat.M8699, Sigma-Aldrich, Germany) was used to inhibit proteasome activity in order to show the proteasome-mediated degradation of BRD4, BRD2, and BRD3 by dBET1. For quantified results shown in Figure 1, densities of protein bands of BRD4 in each cell line were quantified by ImageJ and normalized to the densities of GAPDH bands. Quantified densities of BRD4 were then normalized to that of Kasumi cells and used to represent the relative expression of BRD4.

### Statistics Analysis

All experiments were performed in triplicates and independently repeated at least three times. Statistical analyses were performed using GraphPad Prism (version 8) (GraphPad Software, Inc., San Diego, CA, United States). *p* values were estimated by two-tail *t* test and <0.05 was regarded as statistically significant (*p < 0.05, **p < 0.01, ***p < 0.001, ****p < 0.0001). Error bar was indicated by Means ± Standard Deviation (SD).

## Results

### High Expression of BRD4 Was Associated With Poor Prognosis in Acute Myeloid Leukemia

Recently, our lab [34] reported that high expression of BRD4 in T-ALL cell lines in the Cancer Cell Line Encyclopedia database was associated with poor prognosis of patients with T-ALL in The Cancer Genome Atlas (TCGA) database. Of note, BRD4 was also highly expressed in AML, T-ALL, B-ALL, and B cell lymphoma compared with that in other cancer cell lines (Figure 1A). Taken together, these results signify strong dependence of these cancer cell lines on high expression of BRD4, indicating the potential therapeutic effect of BET family inhibition. Moreover, we explored BRD4 expression in a cohort of 148 adult patients with AML from TCGA database. Patients with AML with high expression of BRD4 have a significantly lower overall survival (OS) rate than those with low expression of BRD4 (*p* = 0.044) (Figure 1B).

### Anti-Cancer Effect of BET Family Inhibition on Acute Myeloid Leukemia Cell Lines

Given its expression in AML cell lines, we hypothesize that survival of AML cell lines might rely on the expression of BRD4 and inhibiting BRD4 might be toxic to AML cell lines. To this end, we first confirmed the expression of BRD4 in four AML cell lines (i.e., NB4, Kasumi, THP-1, and MV4-11) using Western blotting (Figure 1C). BRD4 was expressed in all four cell lines, and its quantified expression is shown in Figure 1D. In addition, the expressions of BRD3 and BRD2 were also confirmed in the same four cell lines (Figure 1E). Then, we performed CCK-8 assay using dBET1, a PROTAC inhibitor targeting BET family to test the cytotoxic effects of inhibiting BET family. Potent cytotoxicity was observed in all four AML cell lines (Kasumi IC_50_: 0.1483 μM, NB4 IC_50_: 0.3357 μM, THP-1 IC_50_: 0.3551 μM, and MV4-11 IC50: 0.2748 μM) (Figure 1F).

### Efficient Targeted-Degradation of the BRD Family by dBET1

**FIGURE 2:**
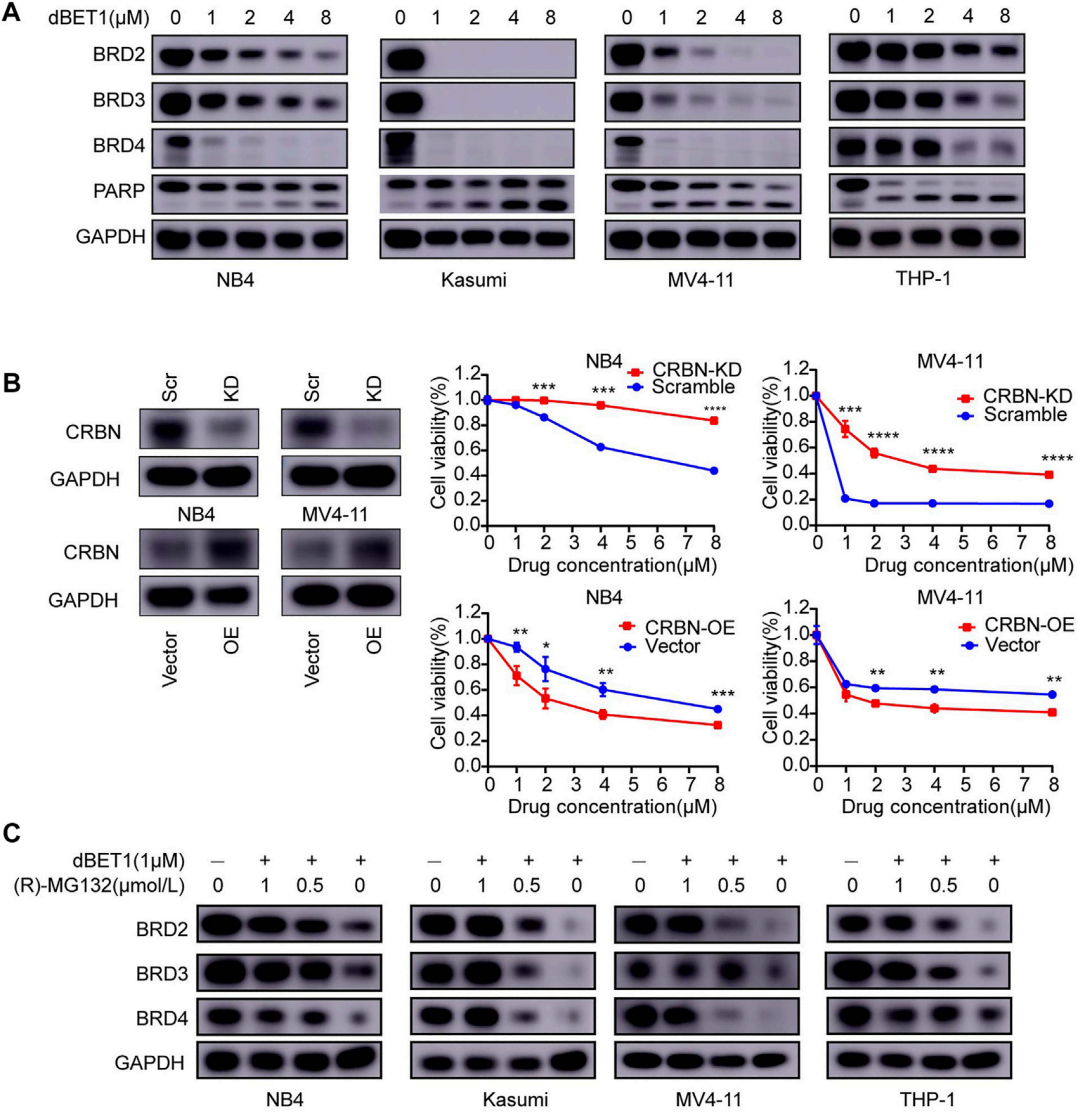
BRD2, BRD3, and BRD4 are specific targets of dBET1 in a CRBN-dependent manner. **(A)** After treatment with dBET1, the expression of BRD2, BRD3, and BRD4 was determined using Western blotting in NB4, Kasumi, MV4-11, and THP-1 cells. Compared with that in the control, the expression of BRD2, BRD3, and BRD4 was decreased in a dose-dependent manner. **(B)** Since dBET1 is a CRBN-based BET degrader, we knocked down CRBN using shRNA and found that downregulation of CRBN significantly increased the resistance of NB4 and MV4-11 cells to dBET1 (top panel). In contrast, overexpression of CRBN increased the sensitivity ofNB4 and MV4-11 cells to dBET1 (bottom panel). **(C)** Since dBET1-mediated degradation of BRD2, BRD3, and BRD4 was through a ubiquitin–proteasome system, we used the proteasome inhibitor, (R)-MG132, which inhibited the degradation of BRD2, BRD3, and BRD4 in a dose-dependent manner. KD: knock down, OE: overexpression, scr: scramble.

To prove that dBET1-mediated degradation of the BRD family (i.e., BRD2, BRD3, and BRD4) has cytotoxic effects on AML cell lines, we determined the protein levels of the BRD family in four AML cell lines treated with increasing concentrations of dBET1. Efficient degradation of the BRD family in a dose-dependent manner was observed in all four cell lines, with the most efficiency observed in Kasumi cells, followed by MV4-11, NB4, and THP-1, which conforms to the IC_50_ values of dBET1 in these four cell lines: Kasumi (0.1483 μM), followed by MV4-11 (0.2748 μM), NB4 (0.3357 μM), and THP-1 (0.3551 μM) (Figure 2A). Besides, concomitant increasing cleavage of PARP was observed in a dose-dependent manner (Figure 2A, bottom panel). Since cereblon (CRBN) acts as an adaptor between BRD family proteins and dBET1, CRBN depletion would compromise the cytotoxic effects of dBET1. To test this hypothesis, we knocked down CRBN using shRNA (Figure 2B, left panel) in NB4 and MV4-11 cells, followed by CCK-8 assay (Figure 2B, right panel). Compared with the scramble control, AML cell lines with knocked down CRBN were significantly more resistant to dBET1; conversely, restoring CRBN expression by CRBN overexpression in AML cell lines with downregulated CRBN reversed the resistance of AML cell lines to dBET1 (Figure 2B, lower panel). Furthermore, to confirm that BRD family proteins are degraded through proteasome-mediated protein degradation, dBET1 treated AML cell lines were maintained with or without the proteasome inhibitor, (R)-MG132. (R)-MG132 sufficiently repressed the degradation of BRD family proteins in a dose-dependent manner (Figure 2C).

### dBET1 Inhibited Cell Proliferation of Acute Myeloid Leukemia Cell Lines

**FIGURE 3:**
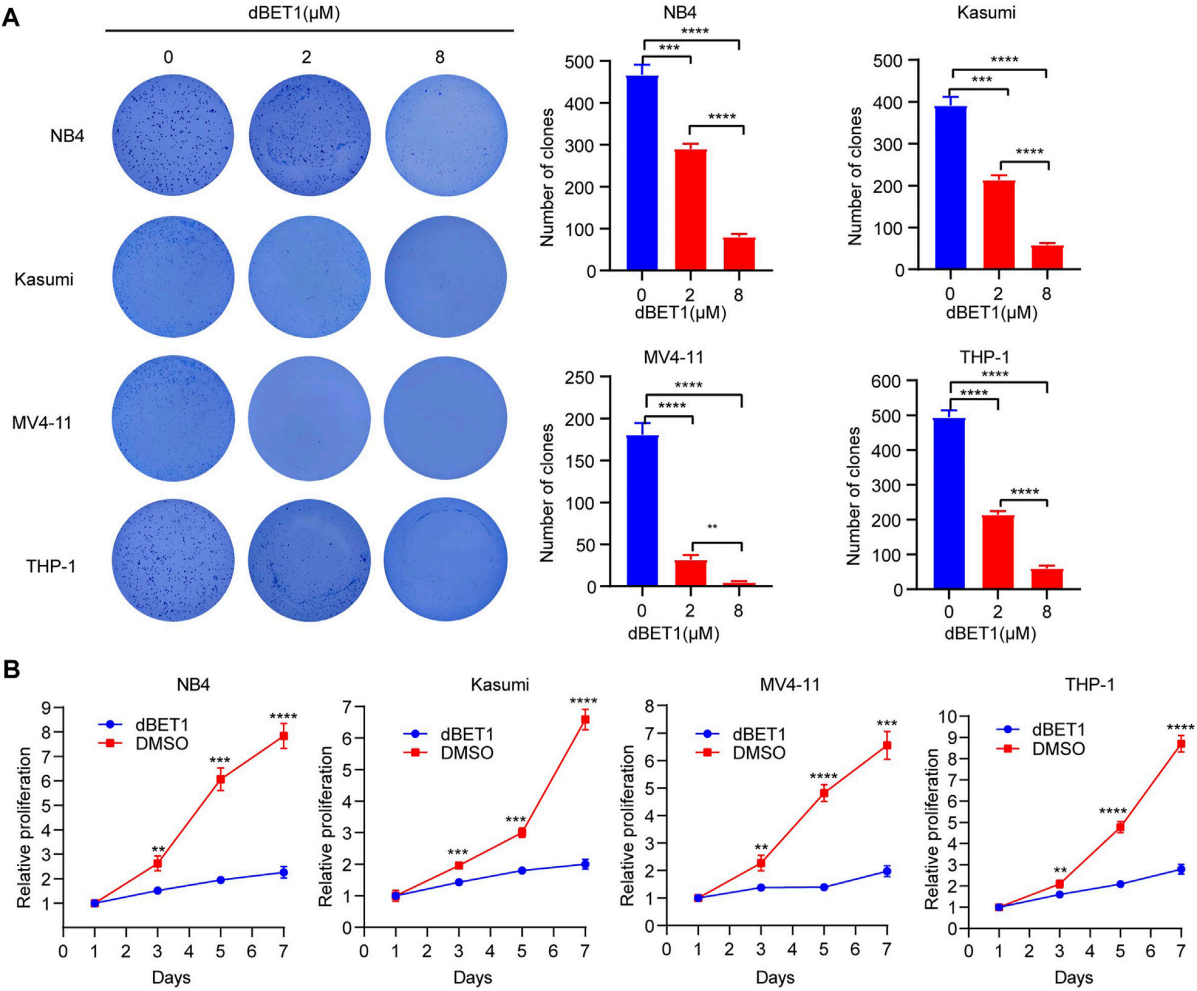
dBET1 inhibited cell proliferation. **(A)** Colony formation assay was performed to characterize the effects of dBET1 on colony formation ability. In the left panel, significantly less colonies were formed in a dose-dependent manner. The quantified results are shown in the right panel. **(B)** Proliferation assay was performed to assess the effects of dBET1 on cell proliferation. Compared with control, dBET1 treatment significantly decreased cell proliferation of NB4, Kasumi, MV4-11, and THP-1 cells.

To characterize the effects of dBET1 on cell proliferation, we conducted soft agar colony formation assay with incremental dosages of dBET1. The numbers of colonies significantly decreased after treatment with dBET1 in a dosage-dependent manner in the four AML cell lines (i.e., NB4, Kasumi, THP-1, and MV4-11) (Figure 3A, left panel). The quantified results were shown in the right panel of Figure 3A. Furthermore, cell proliferation was significantly compromised when treated with dBET1 (1 μM) in these four AML cell lines, compared with those treated with DMSO (Figure 3B). These results indicated that dBET1 significantly inhibited cell proliferation of AML cell lines.

### dBET1 Inhibited the Cell Cycle and Enhanced Apoptosis of Acute Myeloid Leukemia Cell Lines

**FIGURE 4:**
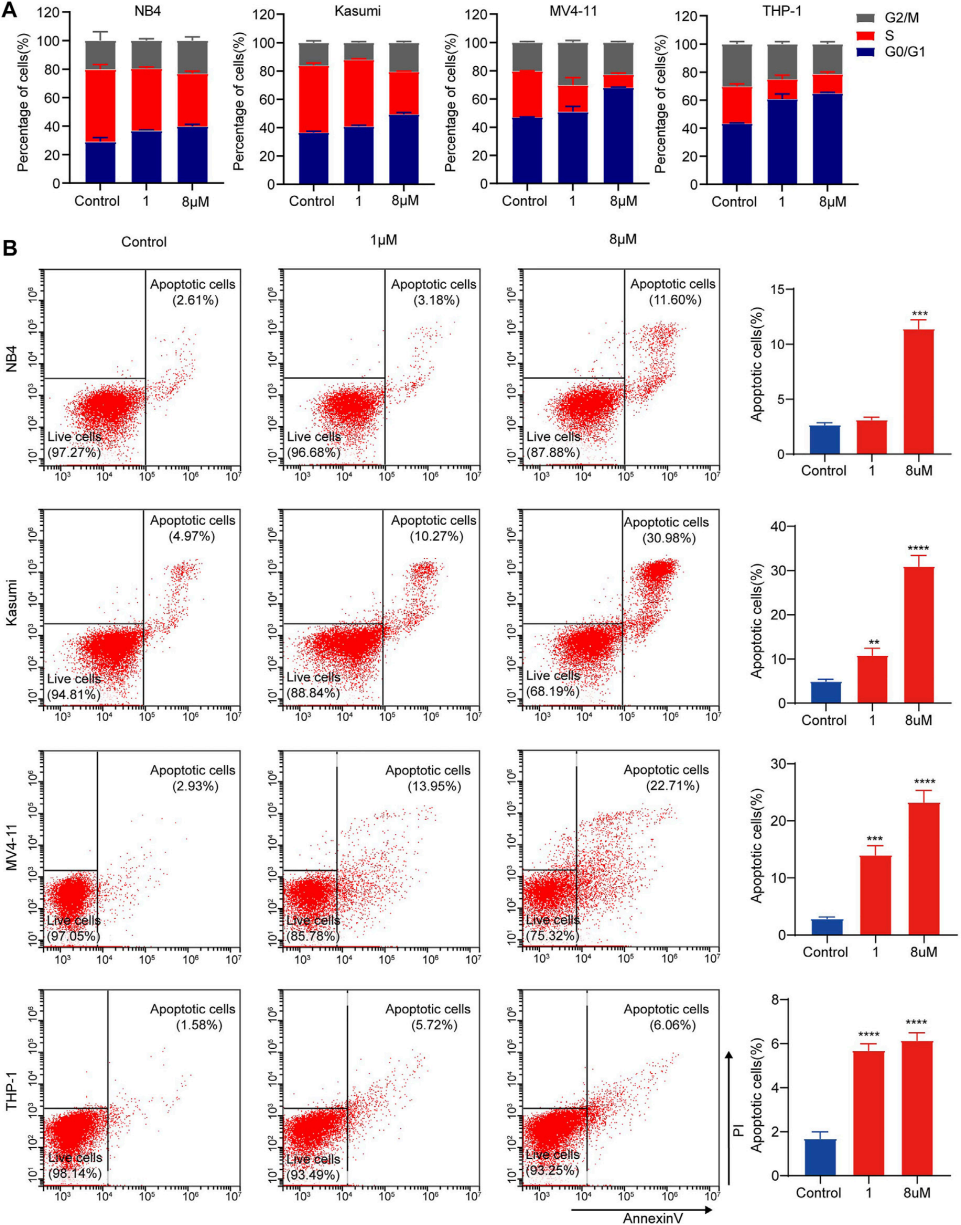
dBET1 arrested cell cycle and enhanced cell apoptosis of AML cell lines. **(A)** Flow cytometry was performed to assess the distribution of cell cycle after staining with PI. The percentages of cells in different phases of cell cycle were compared before and after dBET1 treatment (1 and 8 μM) for 24 h. **(B)** Flow cytometry was performed to determine cell apoptosis after staining with PI and Annexin V. Annexin V positive cells were used to indicate apoptotic cells. Representative flow charts are shown in the left panel and quantitative results are shown in the right panel. Cells were treated with dBET1 (1 and 8 μM) for 24 h.

To further define the effects of dBET1 on cell proliferation, we first assessed cell cycle distribution after dBET1 treatment. Treatment with dBET1 significantly decreased the percentage of cells in the S phase compared with that treated with DMSO control in a dosage-dependent manner. In contrast, the percentage of cells in the GO/G1 phase significantly increased after dBET1 treatment. Collectively, these results showed that inhibiting BET family by dBET1 arrested the cell cycle of the four AML cell lines tested. Moreover, we performed flow cytometry using Annexin V and PI to detect apoptosis at 24 h after dBET1 treatment (1 μM and 8 μM) in the four AML cell lines. Treatment with dBET1 induced more apoptotic cells in a dosage-dependent manner (Figure 4B, left panel). The quantified percentages of apoptotic cells are shown in the right panel of Figure 4B. Collectively, these results suggested that dBET1 led to arrested the cell cycle and enhanced apoptosis in AML cell lines.

### MYC as a Critical Target of dBET1

**FIGURE 5:**
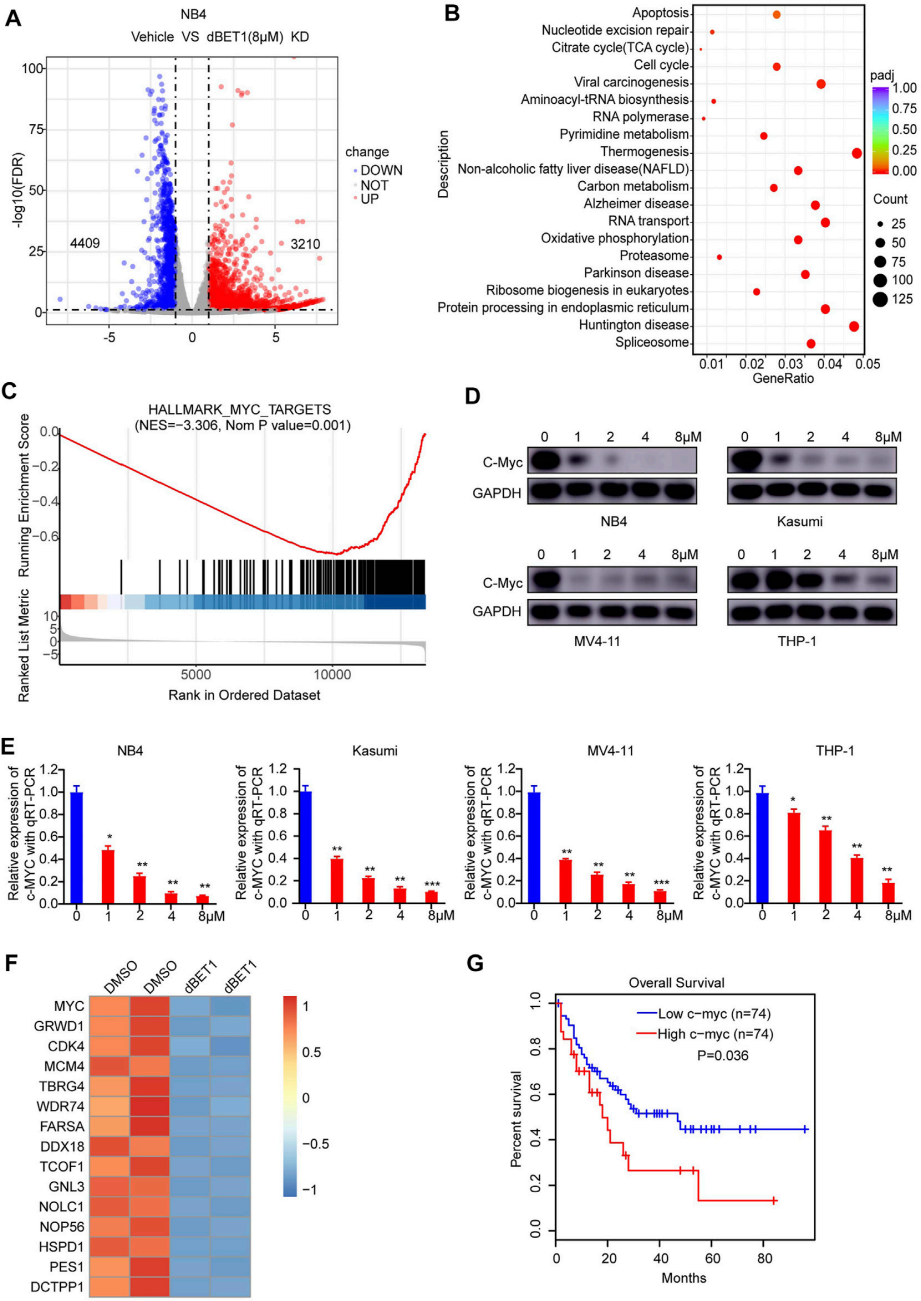
c-MYC was a downstream effector of dBET1. **(A)** After 24 h of treatment with either DMSO or dBET1 (8 μM), total RNA from NB4 cells was purified and subjected to next-generation sequencing. A volcano plot was used to show differentially expressed genes (*p* < 0.001, Fold change >2). **(B)** GO enrichment analysis was performed to show the enriched biological processes. The redder the color, the lower of the *p* value. The bigger the dot, the more enriched the genes. **(C)** Downregulated c-MYC target genes were enriched by GSEA. NES = −3.306, *p* value = 0.001. **(D–E)** dBET1 treatment led to downregulated c-MYC in a dose-dependent manner in NB4, Kasumi, MV4-11, and THP-1 cells, determined using Western blotting **(D)** and qRT-PCR **(E)**. **(F)** Derived from the RNA-seq data, a list of downregulated c-MYC target genes (*p* < 0.05, Fold change >1) were shown. **(G)** The expression of c-MYC was significantly associated with poor survival of adult patients with AML (*n* = 148). *p* = 0.036.

The BRD family plays important roles as a modulator of chromatin remodeling and transcriptional regulation of critical genes in cellular homeostasis and disease progression [35]. Thus, looking into the downstream signaling of the BRD family might provide valuable insights into the underlying mechanism, offering new opportunities for developing better therapies. For this purpose, we compared the transcriptomes of NB4 cells treated with or without dBET1. As a result, 4,409 genes were downregulated, whereas 3,210 genes were upregulated after treatment with dBET1 (Figure 5A volcano plot; Supplementary Data Sheet S1). Then, we performed GO analysis, and apoptosis and cell cycle were among the top enriched biological processes (Figure 5B), which is consistent with the arrested cell cycle and enhanced apoptosis after treatment with dBET1 as shown above. Moreover, downregulated c-MYC target genes were enriched by GSEA (Figure 5C; Supplementary Data Sheet S2). The roles of c-MYC in AML have been extensively studied, and its downregulation would lead to cell cycle arrest and induce apoptosis in AML cell lines [36, 37]. As expected, the expression of c-MYC was downregulated after treatment of dBET1 in a dose-dependent manner in the four AML cell lines, determined by Western blotting and qRT-PCR (Figures 5D,E). In addition to c-MYC, its target genes were also downregulated after dBET1 treatment (Figure 5F). Furthermore, high expression of c-MYC was significantly associated with poor prognosis in a cohort of 148 adult patients with AML from TCGA database (Figure 5G). Taken together, these results highlighted c-MYC as a downstream effector of dBET1 in AML cell lines.

## Discussion

The OS rate of AML is poor compared with those of other acute leukemias. Except for APL and CBF AML, the prognosis of AML remains poor, with only approximately 60% of patients progressing to remission and approximately 30% of patients achieving long-term remission [7, 38]. The risk-adapted therapy with conventional chemotherapeutic agents (daunorubicin + cytarabine) has reached the limit to further improve the treatment outcomes in patients with AML, especially those who are older [7]. Novel therapies are urgently needed to further improve the treatment outcomes of AML. The genetic heterogeneity of AML has been extensively explored and provided several new therapeutic targets. Indeed, numerous novel targeted therapies have recently been developed to fit patients with AML with certain genetic mutations [39, 40]. Among these new agents, either as single agent therapy or a component of combination therapy, only gilteritinib (FLT3 inhibitor) and venetoclax (BCL-2 inhibitor) showed better OS rates [2, 41]. Our results indicated the therapeutic potential of dBET1, a PROTAC inhibitor of BET family members (i.e., BRD2, BRD3, and BRD4) in Kasumi (AML1-ETO), NB4 (PML-RARa), THP-1 (MLL-AF9), and MV4-11 (MLL-AF4) AML cell lines representing four major molecular subtypes of AML. dBET1 is a CRBN-mediated PROTAC through proteasome degradation. Our results confirmed the dependence of dBET1 on CRBN and proteasome (Figures 2B,C).

BRD4 has emerged as a therapeutic target in AML. In this study, we explored the expression of BRD4 across different cancer cell lines and found that BRD4 was highly expressed in AML cell lines, indicating the dependence of AML cell lines on BRD4 for survival and the usage of BRD4 as a potential therapeutic target. Besides, high BRD4 expression was significantly associated with worse OS of adult patients with AML than low BRD4 expression. These results suggested that high BRD4 expression conferred a survival advantage of AML cell lines and represented as a vulnerability. As expected, inhibiting the BET family proteins using dBET1 demonstrated efficient cytotoxic effects on AML cell lines. Previously, dBET1 has shown cytotoxic effects on MV4-11 cells, without knowing its underlying molecular mechanism [28]. In this study, we tested dBET1 in more AML cell lines, aiming to expand its potential therapeutic effects to more molecular subtypes of AML. Furthermore, we showed that dBET1 treatment decreased cell proliferation and arrested cell cycle and enhanced apoptosis of the AML cell lines tested, explaining the molecular mechanism of the high dependence of AML cell lines on the expression of BRD4. Of note, even though BRD4 expression was relatively low in Kasumi cells when compared to other AML cell lines, the depletion of BRD4 as well as BRD2 and BRD3 was more efficient than that in other AML cell lines after treatment of dBET1 (Figure 2A). In line with this, dBET1 shows the lowest IC50 value in Kasumi cells and results in relatively higher apoptosis compared to other AML cell lines.

To further explore the downstream pathways affected by dBET1 treatment, we compared the transcriptomes of AML cell lines with or without dBET1 treatment using RNA-seq. Of note, cell cycle and apoptosis are two enriched pathways because of dBET1 treatment which agrees with the results of the colony formation assay, cell proliferation, cell cycle, and apoptosis analyses. Besides, c-MYC was a well-known downstream target of the BET family, which was also confirmed in this study. Given that c-MYC was an oncogene and played important roles in promoting cell proliferation of cancer cells [42], the downregulation of c-MYC by dBET1 at least partially explained the observed arrested cell cycle, indicating c-MYC as a downstream effector of dBET1’s cytotoxic effects. In line with this, c-MYC target genes were also downregulated.

The BCL6 gene was essential for the survival and self-renewal of AML cell lines, especially those with high expression of BCL6 [43]. Interestingly, BCL6 was the top gene that was upregulated because of dBET1 treatment (data not shown). The upregulation of BCL6 due to dBET1 treatment further suggested the dependence of AML cell lines on high expression of BCL6 [43]. One would reasonably postulate that BCL6 upregulation represents another therapeutic target in AML and provided an opportunity for developing a therapy consisting of a BCL6 inhibitor and dBET1 to achieve better treatment outcomes.

In conclusion, we tested the cytotoxic effects of dBET1 on several AML cell lines representing different molecular subtypes of AML and mechanistically explored the downstream effector of dBET1. Our results revealed the therapeutic potential of dBET1 for AML with different molecular lesions.

## Data Availability

The datasets presented in this study can be found in online repositories. The names of the repository/repositories and accession number(s) can be found in the article/Supplementary Material.

## Supplementary Material

Supplementary Data Sheet S1 Gene Set Enrichment Analysis (GSEA) with Hallmark signatures.

Supplementary Data Sheet S2 RNA-seq data of NB4 cells treated with dBET1 or DMSO for 24 h.

## References

[1] Vardiman JW Thiele J Arber DA Brunning RD Borowitz MJ Porwit A The 2008 Revision of the World Health Organization (WHO) Classification of Myeloid Neoplasms and Acute Leukemia: Rationale and Important Changes. Blood (2009) 114(5):937–51. 10.1182/blood-2009-03-209262 19357394

[2] Dohner H Weisdorf DJ Bloomfield CD. Acute Myeloid Leukemia. N Engl J Med (2015) 373(12):1136–52. 10.1056/nejmra1406184 26376137

[3] Kantarjian H Kadia T DiNardo C Daver N Borthakur G Jabbour E Acute Myeloid Leukemia: Current Progress and Future Directions. Blood Cancer J (2021) 11(2):41. 10.1038/s41408-021-00425-3 33619261 PMC7900255

[4] Kadia TM Ravandi F O'Brien S Cortes J Kantarjian HM. Progress in Acute Myeloid Leukemia. Clin Lymphoma Myeloma Leuk (2015) 15(3):139–51. 10.1016/j.clml.2014.08.006 25441110 PMC4344862

[5] Dohner H Estey EH Amadori S Appelbaum FR Buchner T Burnett AK Diagnosis and Management of Acute Myeloid Leukemia in Adults: Recommendations from an International Expert Panel, on Behalf of the European LeukemiaNet. Blood (2010) 115(3):453–74. 10.1182/blood-2009-07-235358 19880497

[6] Estey EH. Acute Myeloid Leukemia: 2014 Update on Risk-Stratification and Management. Am J Hematol (2014) 89(11):1063–81. 10.1002/ajh.23834 25318680

[7] Tamamyan G Kadia T Ravandi F Borthakur G Cortes J Jabbour E Frontline Treatment of Acute Myeloid Leukemia in Adults. Crit Rev Oncol Hematol (2017) 110:20–34. 10.1016/j.critrevonc.2016.12.004 28109402 PMC5410376

[8] Dovey OM Cooper JL Mupo A Grove CS Lynn C Conte N Molecular Synergy Underlies the Co-occurrence Patterns and Phenotype of NPM1-Mutant Acute Myeloid Leukemia. Blood (2017) 130(17):1911–22. 10.1182/blood-2017-01-760595 28835438 PMC5672315

[9] Ambinder AJ Levis M. Potential Targeting of FLT3 Acute Myeloid Leukemia. Haematologica (2021) 106(3):671–81. 10.3324/haematol.2019.240754 32703795 PMC7927884

[10] Ferret Y Boissel N Helevaut N Madic J Nibourel O Marceau-Renaut A Clinical Relevance of IDH1/2 Mutant Allele burden during Follow-Up in Acute Myeloid Leukemia. A Study by the French ALFA Group. Haematologica (2018) 103(5):822–9. 10.3324/haematol.2017.183525 29472349 PMC5927984

[11] Klein K Kaspers G Harrison CJ Beverloo HB Reedijk A Bongers M Clinical Impact of Additional Cytogenetic Aberrations, cKIT and RAS Mutations, and Treatment Elements in Pediatric T(8;21)-AML: Results from an International Retrospective Study by the International Berlin-Frankfurt-Munster Study Group. J Clin Oncol (2015) 33(36):4247–58. 10.1200/jco.2015.61.1947 26573082 PMC5321085

[12] Wei Y Cao Y Sun R Cheng L Xiong X Jin X Targeting Bcl-2 Proteins in Acute Myeloid Leukemia. Front Oncol (2020) 10:584974. 10.3389/fonc.2020.584974 33251145 PMC7674767

[13] Wong TN Ramsingh G Young AL Miller CA Touma W Welch JS Role of TP53 Mutations in the Origin and Evolution of Therapy-Related Acute Myeloid Leukaemia. Nature (2015) 518(7540):552–5. 10.1038/nature13968 25487151 PMC4403236

[14] Larrosa-Garcia M Baer MR. FLT3 Inhibitors in Acute Myeloid Leukemia: Current Status and Future Directions. Mol Cancer Ther (2017) 16(6):991–1001. 10.1158/1535-7163.mct-16-0876 28576946 PMC5600895

[15] Lam SSY Leung AYH. Overcoming Resistance to FLT3 Inhibitors in the Treatment of FLT3-Mutated AML. Int J Mol Sci (2020) 21(4). 10.3390/ijms21041537 PMC7073218 32102366

[16] Dawson MA Kouzarides T Huntly BJ. Targeting Epigenetic Readers in Cancer. N Engl J Med (2012) 367:7. 10.1056/NEJMra1112635 22894577

[17] Shi J Vakoc CR. The Mechanisms behind the Therapeutic Activity of BET Bromodomain Inhibition. Mol Cel (2014) 54(5):728–36. 10.1016/j.molcel.2014.05.016 PMC4236231 24905006

[18] Roe JS Mercan F Rivera K Pappin DJ Vakoc CR. BET Bromodomain Inhibition Suppresses the Function of Hematopoietic Transcription Factors in Acute Myeloid Leukemia. Mol Cel (2015) 58(6):1028–39. 10.1016/j.molcel.2015.04.011 PMC4475489 25982114

[19] Stathis A Bertoni F. BET Proteins as Targets for Anticancer Treatment. Cancer Discov (2018) 8(1):24–36. 10.1158/2159-8290.cd-17-0605 29263030

[20] Wang N Wu R Tang D Kang R. The BET Family in Immunity and Disease. Sig Transduct Target Ther (2021) 6(1):23. 10.1038/s41392-020-00384-4 PMC7813845 33462181

[21] Baratta MG Schinzel AC Zwang Y Bandopadhayay P Bowman-Colin C Kutt J An In-Tumor Genetic Screen Reveals that the BET Bromodomain Protein, BRD4, Is a Potential Therapeutic Target in Ovarian Carcinoma. Proc Natl Acad Sci U.S.A (2015) 112(1):232–7. 10.1073/pnas.1422165112 25535366 PMC4291641

[22] Toyoshima M Howie HL Imakura M Walsh RM Annis JE Chang AN Functional Genomics Identifies Therapeutic Targets for MYC-Driven Cancer. Proc Natl Acad Sci U.S.A (2012) 109(24):9545–50. 10.1073/pnas.1121119109 22623531 PMC3386069

[23] Zuber J Shi J Wang E Rappaport AR Herrmann H Sison EA RNAi Screen Identifies Brd4 as a Therapeutic Target in Acute Myeloid Leukaemia. Nature (2011) 478(7370):524–8. 10.1038/nature10334 21814200 PMC3328300

[24] Marcotte R Sayad A Brown KR Sanchez-Garcia F Reimand J Haider M Functional Genomic Landscape of Human Breast Cancer Drivers, Vulnerabilities, and Resistance. Cell (2016) 164(1-2):293–309. 10.1016/j.cell.2015.11.062 26771497 PMC4724865

[25] Delmore JE Issa GC Lemieux ME Rahl PB Shi J Jacobs HM BET Bromodomain Inhibition as a Therapeutic Strategy to Target C-Myc. Cell (2011) 146(6):904–17. 10.1016/j.cell.2011.08.017 21889194 PMC3187920

[26] Sun X Gao H Yang Y He M Wu Y Song Y PROTACs: Great Opportunities for Academia and Industry. Sig Transduct Target Ther (2019) 4:64. 10.1038/s41392-019-0101-6 PMC6927964 31885879

[27] Toure M Crews CM. Small-Molecule PROTACS: New Approaches to Protein Degradation. Angew Chem Int Ed Engl (2016) 55(6):1966–73. 10.1002/anie.201507978 26756721

[28] Winter GE Buckley DL Paulk J Roberts JM Souza A Dhe-Paganon S Drug Development. Phthalimide Conjugation as a Strategy for *In Vivo* Target Protein Degradation. Science. Drug Dev (2015) 348(6241):1376–81. 10.1126/science.aab1433 PMC4937790 25999370

[29] Rose D Haferlach T Schnittger S Perglerová K Kern W Haferlach C. Subtype-specific Patterns of Molecular Mutations in Acute Myeloid Leukemia. Leukemia (2017) 31(1):11–7. 10.1038/leu.2016.163 27285584

[30] Lim SL Damnernsawad A Shyamsunder P Chng WJ Han BC Xu L Proteolysis Targeting Chimeric Molecules as Therapy for Multiple Myeloma: Efficacy, Biomarker and Drug Combinations. Haematologica (2019) 104(6):1209–20. 10.3324/haematol.2018.201483 30606790 PMC6545861

[31] Xu L Chen Y Mayakonda A Koh L Chong YK Buckley DL Targetable BET Proteins- and E2F1-dependent Transcriptional Program Maintains the Malignancy of Glioblastoma. Proc Natl Acad Sci U S A (2018) 115(22):E5086–E5095. 10.1073/pnas.1712363115 29764999 PMC5984485

[32] Wu T Hu E Xu S Chen M Guo P Dai Z clusterProfiler 4.0: A Universal Enrichment Tool for Interpreting Omics Data. Innovation (Camb) (2021) 2(3):100141. 10.1016/j.xinn.2021.100141 34557778 PMC8454663

[33] Li Z Yang C Li X Du X Tao Y Ren J The Dual Role of BI 2536, a Small-Molecule Inhibitor that Targets PLK1, in Induction of Apoptosis and Attenuation of Autophagy in Neuroblastoma Cells. J Cancer (2020) 11(11):3274–87. 10.7150/jca.33110 32231733 PMC7097946

[34] Wu S Jiang Y Hong Y Chu X Zhang Z Tao Y BRD4 PROTAC Degrader ARV-825 Inhibits T-Cell Acute Lymphoblastic Leukemia by Targeting 'Undruggable' Myc-Pathway Genes. Cancer Cel Int (2021) 21(1):230. 10.1186/s12935-021-01908-w PMC8061034 33888130

[35] Boyson SP Gao C Quinn K Boyd J Paculova H Frietze S Functional Roles of Bromodomain Proteins in Cancer. Cancers (Basel) (2021) 13(14). 10.3390/cancers13143606 PMC8303718 34298819

[36] Carter JL Hege K Yang J Kalpage HA Su Y Edwards H Targeting Multiple Signaling Pathways: the New Approach to Acute Myeloid Leukemia Therapy. Signal Transduct Target Ther (2020) 5(1):288. 10.1038/s41392-020-00361-x 33335095 PMC7746731

[37] Brondfield S Umesh S Corella A Zuber J Rappaport AR Gaillard C Direct and Indirect Targeting of MYC to Treat Acute Myeloid Leukemia. Cancer Chemother Pharmacol (2015) 76(1):35–46. 10.1007/s00280-015-2766-z 25956709 PMC4485702

[38] Dohner H Estey E Grimwade D Amadori S Appelbaum FR Büchner T Diagnosis and Management of AML in Adults: 2017 ELN Recommendations from an International Expert Panel. Blood (2017) 129(4):424–47. 10.1182/blood-2016-08-733196 27895058 PMC5291965

[39] Yu J Jiang PYZ Sun H Zhang X Jiang Z Li Y Advances in Targeted Therapy for Acute Myeloid Leukemia. Biomark Res (2020) 8:17. 10.1186/s40364-020-00196-2 32477567 PMC7238648

[40] Cucchi DGJ Polak TB Ossenkoppele GJ Uyl–De Groot CA Cloos J Zweegman S Two Decades of Targeted Therapies in Acute Myeloid Leukemia. Leukemia (2021) 35(3):651–60. 10.1038/s41375-021-01164-x 33589753

[41] Levis M Perl AE. Gilteritinib: Potent Targeting of FLT3 Mutations in AML. Blood Adv (2020) 4(6):1178–91. 10.1182/bloodadvances.2019000174 32208491 PMC7094008

[42] Ahmadi SE Rahimi S Zarandi B Chegeni R Safa M. MYC: a Multipurpose Oncogene with Prognostic and Therapeutic Implications in Blood Malignancies. J Hematol Oncol (2021) 14(1):121. 10.1186/s13045-021-01111-4 34372899 PMC8351444

[43] Kawabata KC Zong H Meydan C Wyman S Wouters BJ Sugita M BCL6 Maintains Survival and Self-Renewal of Primary Human Acute Myeloid Leukemia Cells. Blood (2021) 137(6):812–25. 10.1182/blood.2019001745 32911532 PMC7885821

